# Diagnostic accuracy of controlled attenuation parameter (CAP) as a non-invasive test for steatosis in suspected non-alcoholic fatty liver disease: a systematic review and meta-analysis

**DOI:** 10.1186/s12876-019-0961-9

**Published:** 2019-04-08

**Authors:** Ke Pu, Yuping Wang, Suyang Bai, Hui Wei, Yongning Zhou, Jiangao Fan, Liang Qiao

**Affiliations:** 1 Department of Gastroenterology, The First Hospital of Lanzhou University, Lanzhou, China; 2 Key Laboratory for Gastrointestinal Diseases of Gansu Province, Lanzhou University, Lanzhou, China; 3 Storr Liver Centre, Westmead Institute for Medical Research, University of Sydney at Westmead Clinical School, Westmead, Australia

**Keywords:** Controlled attenuation parameter (CAP), Non-alcoholic fatty liver disease (NAFLD), Hepatic steatosis, diagnostic accuracy, Transient elastography

## Abstract

**Background:**

Controlled attenuation parameter (CAP) is a non-invasive method for diagnosing hepatic steatosis. Despite good diagnostic performance, clinical application of CAP is limited due to the influences of covariates. Here, a systematic review on the performance of CAP in the diagnosis and staging of hepatic steatosis in NAFLD patients was performed.

**Methods:**

The sensitivity, specificity, diagnostic odds ratio (DOR) and area under receiver operating characteristics (AUROC) curves of the pooled data for CAP in diagnosing and staging the mild (Stage 1), moderate (Stage 2) and severe (Stage 3) steatosis in NAFLD patients were assessed. The clinical utility of CAP was evaluated by Fagan plot. Heterogeneity was explored using subgroup analysis.

**Results:**

Nine studies involving 1297 patients with liver biopsy-proven NAFLD were analyzed. The pooled sensitivity of CAP in detecting mild hepatic steatosis was 87% with a specificity of 91% and a DOR of 84.35. The pooled sensitivity of CAP in detecting moderate hepatic steatosis was 85% with a specificity of 74% and a DOR of 21.28. For severe steatosis, the pooled sensitivity was 76% with a specificity of 58% and a DOR of 4.70. The mean AUROC value for CAP in the diagnosis of mild, moderate, and severe steatosis was 0.96, 0.82 and 0.70, respectively. A subgroup analysis indicated that variation in the geographic regions, cutoffs, age and body mass index (BMI) could be the potential sources of heterogeneity in the diagnosis of moderate to severe steatosis.

**Conclusions:**

CAP should be cautiously considered as a non-invasive substitute for liver biopsy in clinical practice.

**Electronic supplementary material:**

The online version of this article (10.1186/s12876-019-0961-9) contains supplementary material, which is available to authorized users.

## Background

Non-alcoholic fatty liver disease (NAFLD) is increasingly becoming a serious clinical concern owing to its severe morbidity and potential progression to end stage of liver disease such as liver cirrhosis, and hepatocellular carcinoma (HCC) [1]. The current global prevalence of NAFLD is estimated to be 25.24% [2]. The incidence of NAFLD varies with geographic regions with the highest prevalence in the Middle East and South America and lowest in Africa [2]. In China, there is a striking difference in the prevalence of NAFLD between the East and Central China (38.17%) and the relatively undeveloped West China (12.5%) [3–6]. NAFLD is associated with increased risk of HCC (0.44 per 1000 person-years) and the risk ratios for liver-specific and overall mortality are 1.94 and 1.05, respectively [2]. Hence, NAFLD constitutes a serious health concern and as such, a reliable diagnostic or screening algorithm for NAFLD is needed. Abnormalities in serum liver enzymes are an important part of the routine clinical tests for patients with NAFLD, however, they are affected by multiple factors including obesity, metabolic syndrome, diabetes, as well as ethnic and genetic backgrounds [7]. Moreover, liver enzymes can be normal in the NAFLD patients incidentally detected by ultrasound during routine medical checkups [8–10]. Liver biopsy is often regarded as the reference standard for the diagnosis of NAFLD and for the assessment of NAFLD-associated pathological conditions such as the degree of steatosis and liver fibrosis [9, 11]. However, liver biopsy has well-recognized drawbacks such as invasiveness, adverse events and sampling variability [12], and the histopathological parameters of NAFLD may change following physical activities and therapeutic interventions [13], liver biopsy in NAFLD patients often provides unstable results. Thus, non-invasive diagnostic strategies including serum biomarkers and imaging techniques (e.g., ultrasonography, computerized tomography, magnetic resonance, and ultrasonography-based elastography) have practical advantages in the assessment of NAFLD.

Ultrasound remains the first-line assessment for screening NAFLD patients in clinical practice, but its efficacy is limited by its impreciseness observed during follow-up [14]. Computerized tomography (CT) scan also has shown limited benefit in the diagnosis and follow-up of NAFLD patients [15]. Magnetic resonance imaging (MRI) based-techniques are sensitive approaches for the detection of steatosis due to their specific signal intensity for triglyceride [16], but they are not suitable for routine usage owing to the high cost, limited availability, and limited comparability between different MRI techniques [17, 18]. Thus, non-invasive imaging techniques that can accurately discriminate different stages of hepatic steatosis are highly desirable.

Vibration-controlled transient elastography (VCTE) commonly delivered by the FibroScan device (Echosens, Paris, France) measures the velocity of the shear wave that is converted to stiffness using the Young’s module [19]. It has been recognized as a rapid and non-invasive technique in the diagnosis and staging of liver fibrosis [20–23]. Controlled attenuation parameter (CAP) is a novel physical parameter based on the properties of ultrasonic signals acquired by the FbroScan [24]. CAP measures ultrasound attenuation at the central frequency of the VCTE at M or regular probe [25], but its accuracy may be affected by variations in cut-off values of different steatosis grades and different covariates [26].

In this study, we analyzed the diagnostic accuracy of CAP in distinguishing different stages of hepatic steatosis in liver-biopsy proven NAFLD patients, and assessed the possible contributing factors affecting CAP values.

## Methods

### Study selection

All relevant articles on the application of CAP in the diagnosis of hepatic steatosis in liver-biopsy proven NAFLD patients available on multiple electronic databases including PubMed, EMBASE, Web of Science, and Cochrane Library up to May 12,017 were searched. Heading terms and key words used in the search include “controlled attenuation parameter” or “CAP”, “hepatic steatosis” or “liver steatosis” or “steatohepatitis”, “non-alcoholic fatty liver disease” or “NAFLD”, “diagnosis accuracy” or “diagnostic test”. The references screened by titles and abstracts therein were firstly reviewed by two authors (KP and SYB) independently and blindly. The remaining articles were further selected by reading full-text to exclude the irrelevant information, as set out below in the inclusion criteria. Only articles published in English were searched.

### Eligibility criteria

Studies were included if the following criteria were met: (1) that performed in NAFLD patients diagnosed by liver biopsy and the degree of fatty liver changes classified as follows: Stage 0 (S0), fatty changes seen in < 5% of hepatocytes; Stage 1 (S1), fatty changes occurred in 5–33% of hepatocytes; Stage 2 (S2), fatty changes seen in 34–66% of hepatocytes; and Stage 3 (S3), > 66% of hepatocytes had fatty changes [27]; (2) that provided adequate description of CAP using transient elastography (FibroScan FS); (3) that liver biopsy was used as the reference standard of the assessment of hepatic steatosis; and (4) that sufficient data were available for calculating the test performance parameters including true positive (TP), false positive (FP), true negative (TN) and false negative (FN) rates. All data were extracted from the primary articles or acquired directly from the corresponding authors. Inclusion was not restricted by study size or publication type.

### Data extraction and quality assessment

From each study included in this analysis, the following data were extracted: primary author; journal and year of publication; country where the study was performed; age, gender, and number of patients; body mass index (BMI); cutoff values; area under the curve (AUC); and study design. Tables containing TP, TN, FP, and FN rates were extracted from the sensitivity and specificity of CAP. Quality assessment of the studies included in this analysis was conducted by two authors independently using the Quality Assessment of Diagnostic Accuracy Studies (QUADAS-2) [28], which consists of four domains including patient selection, index test, reference standard and flow and timing domain. Each signalling question was judged as “yes” or “no” or “unclear”, and the risk of bias and concern for applicability in each study were estimated as “high” or “low”, or “unclear”, except for the flow and timing domain where applicability concern does not apply.

### Statistical analysis

Statistic analyses were performed using the pooled data unless otherwise stated. To evaluate the CAP performance for different stages of hepatic steatosis in NAFLD patients, pooled sensitivity, specificity, likelihood ratio (LR), DOR and AUSROC with standard errors (SE) and Q indexes with SE were analyzed by Meta-Disc Software (Version1.4) using the TP, FP, TN and FN values from the original papers. The sensitivity and specificity provided by the original studies were used to recalculate the above values if sufficient information could be extracted from the source studies, and the summary statistics were presented using the diagnostic threshold effect analyzed by Spearman correlation coefficient and *P*-value. If there was no significant threshold effect, the diagnostic accuracy was estimated by pooled statistics. In this case, the diagnostic accuracy was only evaluated by AUSROC and Q indexes rather than sensitivity, specificity and DOR.

DOR represents the odds of positive CAP in NAFLD patients with steatosis as compared to normal subjects. AUSROC value of 0.5–0.7, 0.7–0.9 and 0.9–1.0 suggests low, moderate and high diagnostic accuracy, respectively. A smaller Q index indicates a lower diagnostic accuracy.

A positive LR (PLR) was the probability of a NAFLD patient who had a positive CAP divided by the probability of a non-NAFLD person who had positive CAP [i.e., PLR = sensitivity/(1-specificity)]. A negative LR (NLR) was the probability of NAFLD patient who had a negative CAP divided by the probability of a non-NAFLD person who had a negative CAP [i.e., NLR = (1-sensitivity)/specificity)]. A PLR > 5.0 and NLR < 0.2 suggest a higher diagnostic evidence [29].

Post-test probability was calculated by Fagan’s plot analysis under the presumed condition of pre-test probability of 25, 50 and 75%, respectively, following the positive and negative CAP measurements [30]. This allowed the determination of the relationship between the prior specified probability (Ppre), the LR, and posterior test probability (Ppost). The post-test probability was calculated using the Bayes Theorem: Ppost = (LR × Ppre)/[(1-Ppre) × (l-LR)] [31]. Positive CAP results were defined as all results above the optimal cut-off value for S1, S2, or ≥ S3 given in each individual study, and negative test results refer to all results below the same cut-off values.

Heterogeneity was evaluated by Cochran’s Q Statistic and *I*^*2*^ Statistic with Chi-squared test and Inconsistency test, respectively. The Cochran’s Q Statistic of homogeneity was measured on the basis of the null hypothesis that all eligible studies have the same underlying magnitude of effect [32]. As this test is incapable of detecting moderate degrees of heterogeneity, a *P* value of < 0.10 was considered to show significant heterogeneity;^32^and the *I*^*2*^ Statistic (*I*^*2*^ value) was calculated on the basis of algorithm which estimates proportion of total variation across inclusive studies caused by heterogeneity rather than sampling error. *I*^*2*^ values of 0–40%, 40–70% and 70–100% indicate low, moderate and high variance, respectively [33].

If lower heterogeneity existed or homogeneity in clinical characteristics was noted, the Mantel-Haenszel method was chosen in case of fixed-effects model. Otherwise, the random-effects model was used with DerSimonian Laird method [34]. If an *I*^*2*^ > 50% and/or a *P* < 0.05 was found, considerable heterogeneity was considered, and in this case, sources of heterogeneity were explored by a subsequent subgroup analysis to identify the potential covariates.

Deek’s Funnel Plot was applied to examine the potential publication bias caused by asymmetry of the tests. This analysis uses a regression of the diagnostic logarithm of odds ratio (OR) against 1/sqrt (effective sample size, ESS) and is weighted by ESS. A *P* value of < 0.05 for the slope coefficient indicates test asymmetry and suggests a significant publication bias [33].

Meta-Disc Version 1.4 (Ramon y Cajal Hospital, Madrid, Spain) software was used to generate forest plot and Stata12.0 (Stata Corp, College Station, TX, USA) was used to perform the subgroup analysis, sensitivity analysis and publication bias with the MIDAS and METANDI modules.

## Results

### Study selection and characteristics

**Fig. 1:**
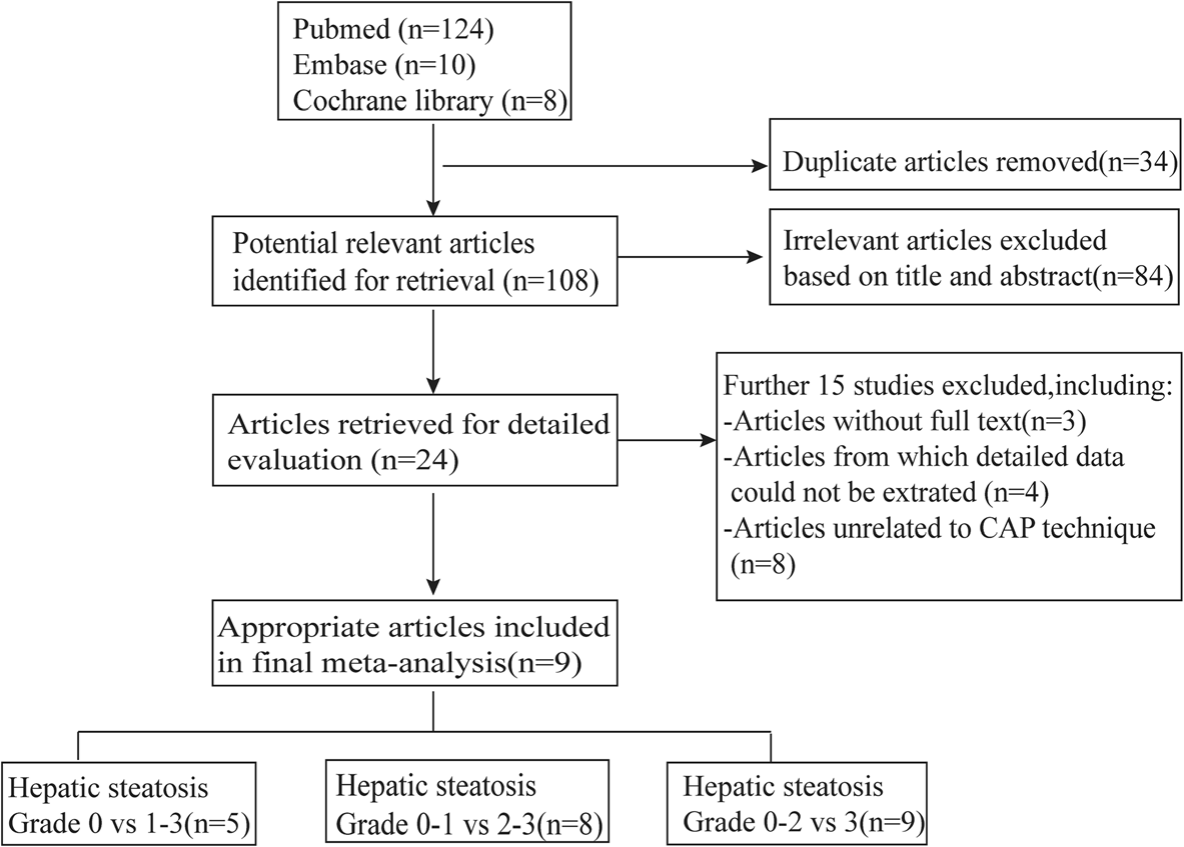
Article selection process

Selection process is presented in Fig. 1. Of the 142 articles searched, 118 were excluded due to duplication (*n* = 34) and irrelevance (*n* = 84) following title and abstract screening. The remaining 24 potentially eligible reports were further evaluated. After excluding the articles with irrelevant contents and articles with no full-text and insufficient data, nine English papers [35–43] were included for the final meta-analysis. In these nine papers, five discussed NAFLD patients with S1 steatosis, eight discussed NAFLD patients with S2 steatosis, and all nine papers discussed NAFLD patients with ≥S3 steatosis. These nine studies involving 1297 NAFLD patients were performed in different geographic regions including Europe (*n* = 3), Asia (*n* = 4), USA (*n* = 1) and multicenter (n = 1). In these studies, the diagnosis and grading for hepatic steatosis were assessed by CAP based on the data obtained from VCTE of the FbroScan at M probe around three months of liver biopsy. Detailed information of these studies is presented in Additional file 1: Table S1 and Table S2.

All patients underwent clinical and biochemical evaluations, and had CAP to assess the level of hepatic steatosis. All patients had stable disease and were without any chronic complications. In the assessment of etiology for NAFLD, long-term alcohol intake and evidence of secondary causes of hepatic steatosis including viral hepatitis, human immunodeficiency virus infection, autoimmune hepatitis, and genetic liver diseases were excluded. Diagnosis of NAFLD, grading of steatosis, inflammation and fibrosis was confirmed by liver biopsy.

**Fig. 2:**
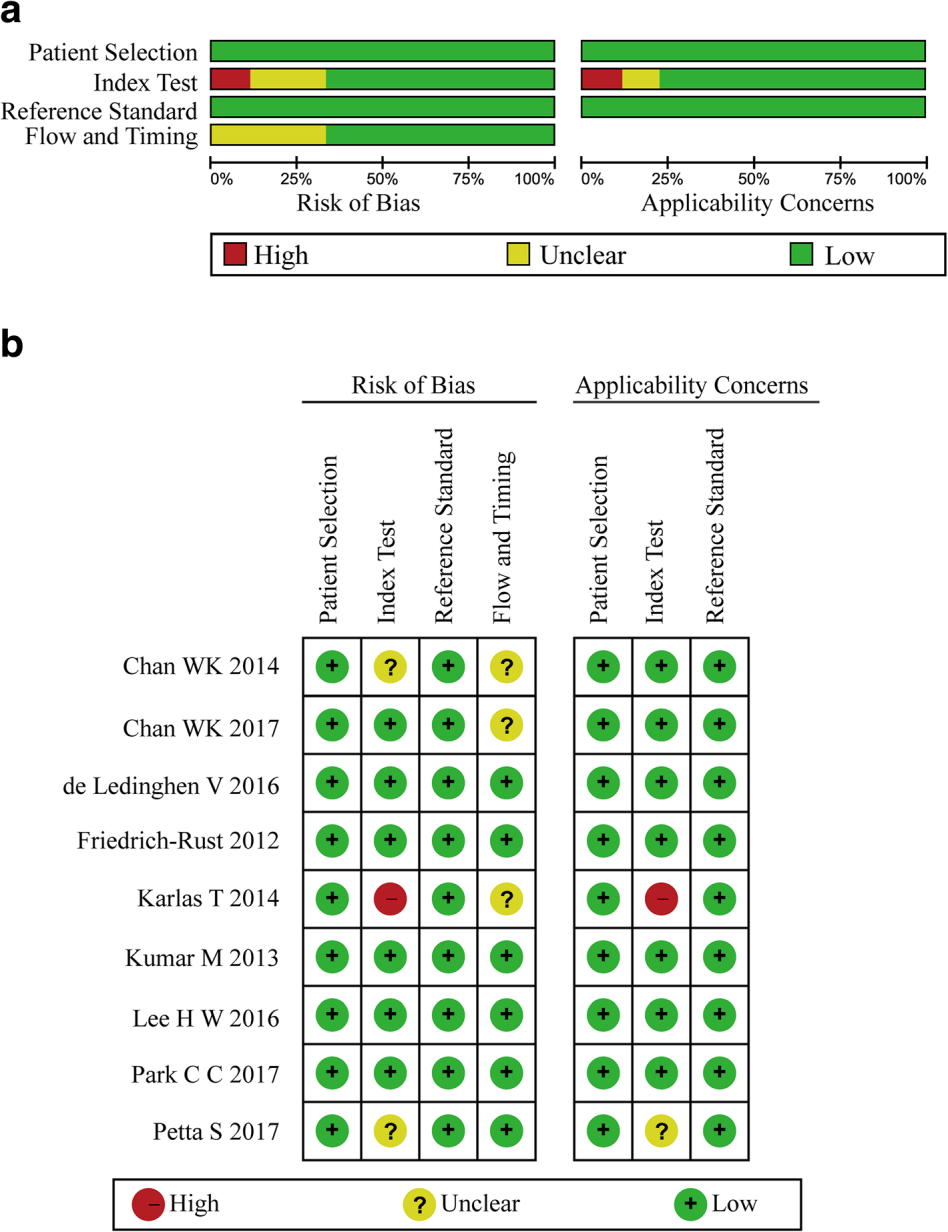
Quality assessment of the included studies by methodological quality graph (**a**) and Cochrane Handbook (**b**)

Histopathological findings were reported as published, and the NAFLD activity score represents the sum of the scores for hepatic steatosis, lobular inflammation and hepatocyte ballooning [27]. Steatosis was graded according to the percent of the affected hepatocytes: mild (S1, 5–33%), moderate (S2, 34–66%), and severe (S3, ≥67%) [27]. The quality of the eligible studies, as assessed by the QUADAS-2 criteria, was independently appraised by two researchers (KP and SYB) (Fig. 2a and b). Two studies were assessed as “high risk” for index test and flow and timing in Risk of Bias. The remaining studies were estimated as “suboptimal” for unclear risk in the following domains: patient selection, index test, flow and timing. Most of the studies were identified as having low bias risk for patient selection and reference standard.

### Diagnostic yield of CAP for hepatic steatosis and grading

**Fig. 3:**
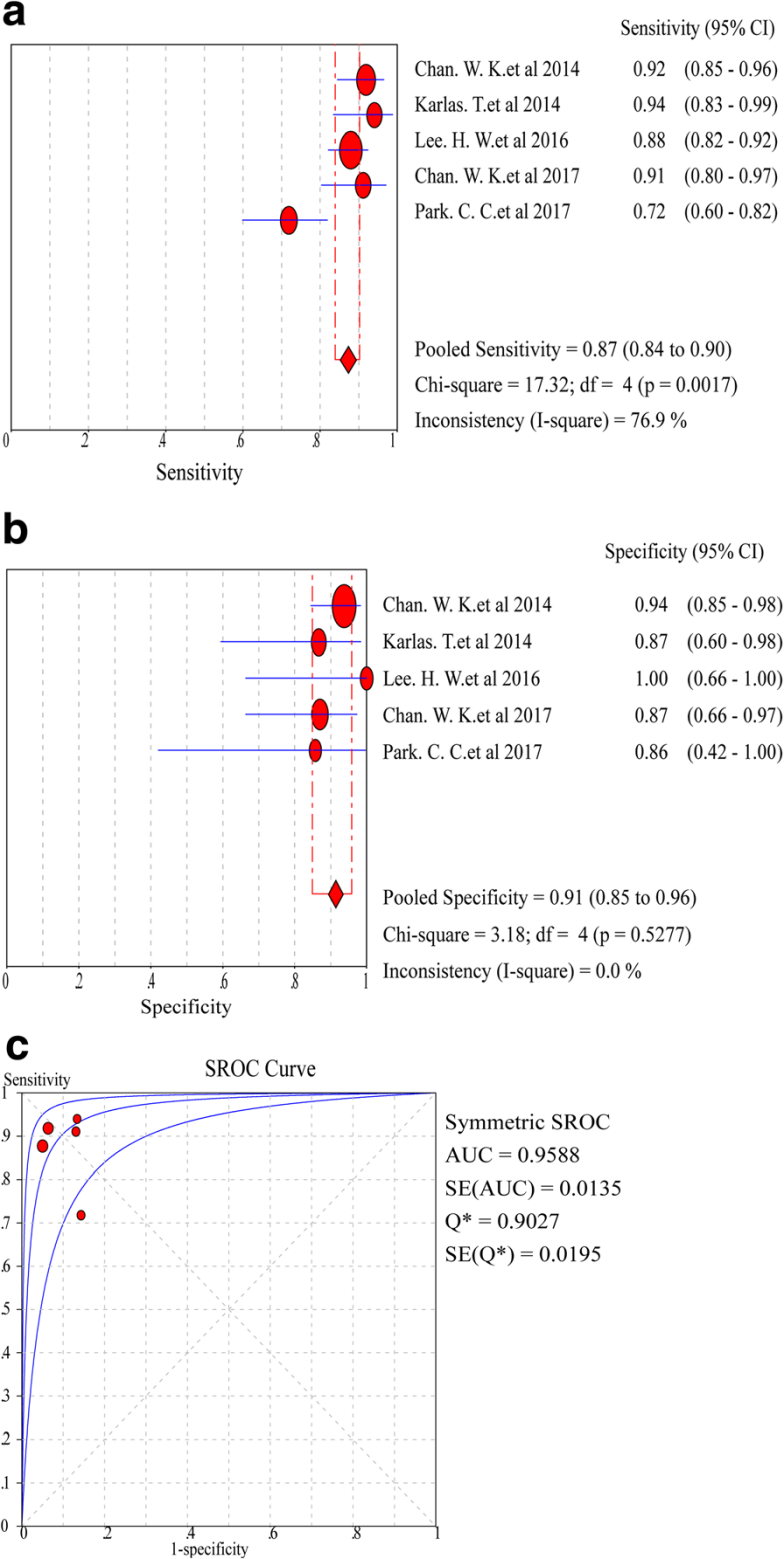
Forest plots and meta-analyses of studies showing pooled sensitivity (**a**) and specificity (**b**) of CAP for detection of ≥S1 steatosis (Stage 0 vs Stage 1–3) in NAFLD patients. **c** Summary of AUROC of CAP for the diagnosis of ≥S1 steatosis (Stage 0 vs Stage 1–3) in NAFLD patients

In the assessment of diagnostic yield for CAP in patients with S1 steatosis, the Cochran-Q statistic and *I*^*2*^ statistic of DOR were 3.71 and 0% (*P* = 0.4463) (Additional file 1: Figure S1, A), indicating no heterogeneity in the included articles. Hence, fixed-effects model was used to generate the pooled effect size. As a result, the pooled sensitivity of five studies was 87% (95% CI: 84.0–90.0%, *I*^*2*^ statistic 76.9%), and the pooled specificity was 91% (95% CI: 85.0–96.0%, *I*^*2*^ statistic 0.0%) (Fig. 3a, b). The pooled DOR was 84.35 (95% CI: 38.35–185.53) (Additional file 1: Figure S1, A), and the pooled AUROC was 0.9588 (SE 0.0135) (Fig. 3c).

**Fig. 4:**
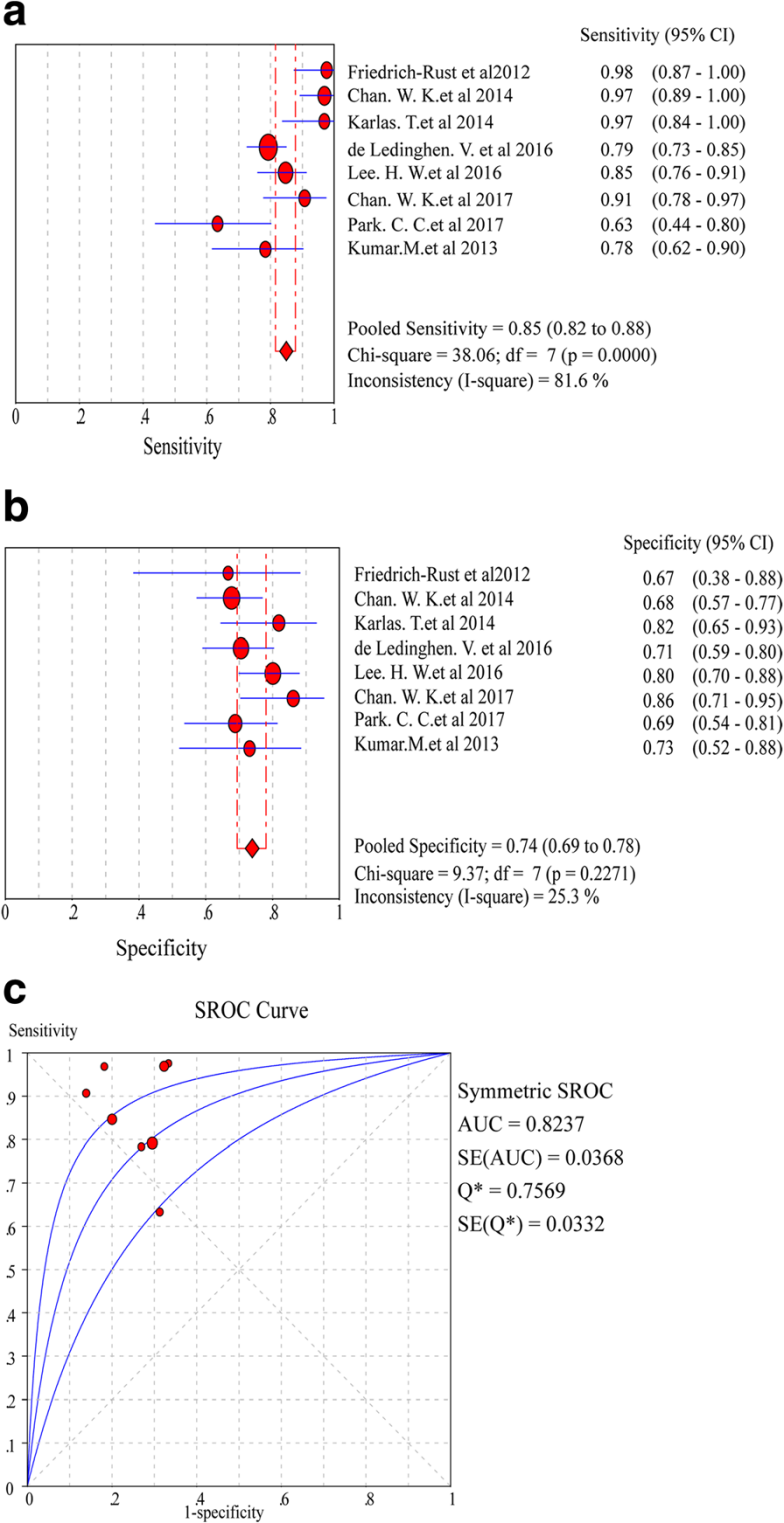
Forest plots and meta-analyses of studies showing pooled sensitivity (**a**) and specificity (**b**) of CAP for detection of ≥S2 steatosis (Stage 0–1 vs Stage 2–3) in NAFLD patients. **c** Summary of AUROC of CAP for the diagnosis of ≥S2 steatosis (Stage 0–1 vs Stage 2–3) in NAFLD patients

In the assessment of diagnostic yield for CAP in patients with ≥S2 steatosis, the Cochran-Q statistic and *I*^*2*^ statistic of DOR were 26.22 and 73.3% (*P* = 0.0005) (Additional file 1: Figure S1, B), indicating a significant heterogeneity between the studies necessitating the selection of a random-effects model for analysis. As a result, the pooled sensitivity of eight studies was 85% (95% CI: 82.0–88.0%, *I*^*2*^ statistic 81.6%), and the pooled specificity was 74% (95% CI: 69.0–78.0%, *I*^*2*^ statistic 25.3%) (Fig. 4a, b). The pooled DOR was 21.28 (95% CI: 9.72–46.57) (Additional file 1: Figure S1, B), and the pooled AUROC was 0.8237 (SE 0.0332) (Fig. 4c). Significant heterogeneity was found in the analysis of eight studies assessing the steatosis grade.

**Fig. 5:**
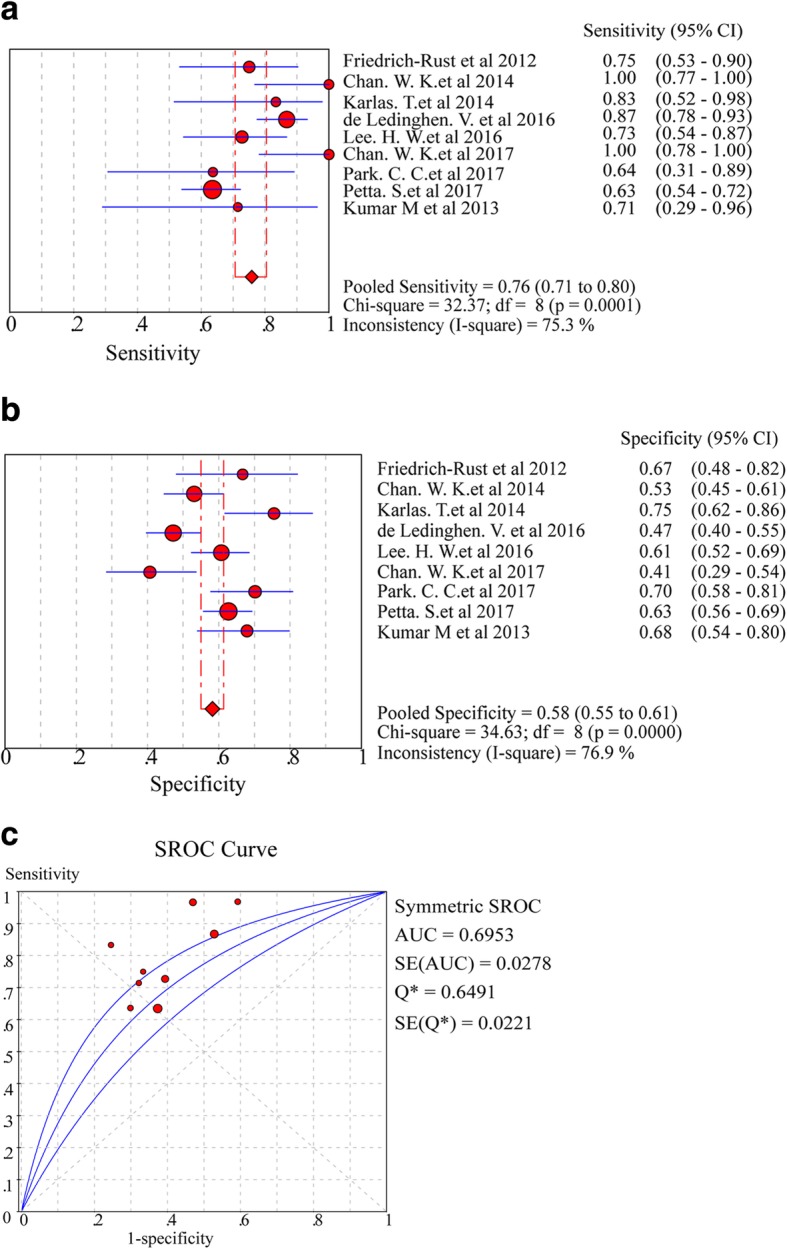
Forest plots and meta-analyses of studies showing pooled sensitivity (**a**) and specificity (**b**) of CAP for detection of ≥S3 steatosis (Stage 0–2 vs Stage 3) in NAFLD patients. **c** Summary of AUROC of CAP for the diagnosis of ≥S3 steatosis (Stage 0–2 vs Stage 3) in NAFLD patients

In the assessment of diagnostic yield for CAP in nine studies with ≥S3 steatosis, the Cochran-Q and *I*^*2*^ statistic of DOR were 9.51 and 15.9% (*P* = 0.3013) (Additional file 1: Figure S1, C), indicating a low heterogeneity in the included articles. Hence, fixed-effects model was used to merge effect size. Of note, the pooled sensitivity of nine studies was 76% (95% CI: 71.0–80.0%, *I*^*2*^ statistic 75.3%), and the pooled specificity was 58% (95% CI: 55.0–61.0%, *I*^*2*^ statistic 76.9%) (Fig. 5a, b). The pooled DOR was 4.71(95% CI:3.54 to 6.27) (Additional file 1: Figure S1, C), and the pooled AUROC was 0.6953 (SE 0.0221) (Fig. 5c).

### Clinical utility of CAP for hepatic steatosis in suspected NAFLD patients

In suspected NAFLD patients with ≥S1steatosis, the Fagan plot analysis revealed a positive and negative likelihood ratio (LR) of 10 and 0.13, respectively. Thus, in this group of patients with 25% pre-test probability (based on clinical suspicion), a positive CAP value revealed a 77% probability of correct diagnosis and a negative CAP value revealed a 4% probability of wrong diagnosis (Additional file 1: Figure S2, A). When the pre-test probability (based on clinical suspicion) was set to 50%, a positive CAP value yielded 91% probability of correct diagnosis and a negative CAP yield a 11% probability of wrong diagnosis (Additional file 1: Figure S2, B). When the pre-test probability (based on clinical suspicion) was set to 75%, a positive CAP value showed 97% probability of correct diagnosis and a negative CAP value showed a 28% probability of wrong diagnosis (Additional file 1: Figure S2, C).

In suspected NAFLD patients with ≥S2 steatosis, the Fagan plot analysis revealed a positive and negative likelihood ratio (LR) of 3 and 0.15, respectively. Thus, in this subset of patients with 25% pre-test probability (based on clinical suspicion), a positive CAP value represented a 53% probability of correct diagnosis and a negative CAP value indicated a 5% probability of wrong diagnosis (Additional file 1: Figure S3, A). When the pre-test probability (based on clinical suspicion) was set to 50%, a positive CAP value showed a 77% probability of correct diagnosis and a negative CAP value showed 13% probability of wrong diagnosis (Additional file 1: Figure S3, B). When the pre-test probability (based on clinical suspicion) was set to 75%, a positive CAP value showed 91% probability of correct diagnosis and a negative CAP value showed a 31% probability of wrong diagnosis (Additional file 1: Figure S3, C).

In suspected NAFLD patients with ≥S3 steatosis, the Fagan plot analysis revealed a positive and negative likelihood ratio (LR) of 2 and 0.37, respectively. Thus, in this subset of patients with 25% pre-test probability (based on clinical suspicion), a positive CAP value represented a 39% probability of correct diagnosis and a negative CAP value indicated a 11% probability of wrong diagnosis (Additional file 1: Figure S4, A). When the pre-test probability (based on clinical suspicion) was set to 50%, a positive CAP value showed a 66% probability of correct diagnosis and a negative CAP value showed a 27% probability of wrong diagnosis (Additional file 1: Figure S4, B). When the pre-test probability (based on clinical suspicion) was set to 75%, a positive CAP value showed 85% probability of correct diagnosis and a negative CAP value showed a 53% probability of wrong diagnosis (Additional file 1: Figure S4, C).

Fagan analysis revealed a pooled positive LR of CAP of 3.00 for ≥S2 steatosis and 2.00 for ≥S3 steatosis, and a pooled negative LR of CAP of 0.15 for ≥S2 steatosis and 0.37 for ≥S3steatosis, respectively. These data translated into a 3-fold and 2-fold higher accuracy of a positive CAP in the diagnosis of ≥S2 and ≥ S3 steatosis in suspected NAFLD patients than in the normal objects, and a lower diagnostic ability for ≥S2 and ≥ S3 steatosis than for ≥S1 steatosis. In patients with a pre-test probability of 25%, no more than 60% of cases with ≥S2 and ≥ S3 steatosis were correctly diagnosed by a positive CAP, and more than 10% of the patients with severe steatosis were missed by a negative CAP. Less than 80% of ≥S2 and ≥ S3 steatosis in suspected NAFLD patients with 50% pre-test probability were correctly diagnosed by CAP, while more than 25% of the patients with severe steatosis were missed by a negative CAP. In suspected NAFLD patients with a pre-test probability of 75%, no more than 90% of the cases with ≥S3 steatosis were correctly diagnosed, while 28–53% of the patients with all stages of steatosis were missed by a negative CAP.

### Subgroup analysis and sensitivity analysis

Given the aforementioned statistically significant heterogeneity of DOR for patients with ≥S2 steatosis (*I*^*2*^ = 73.3%, *P* = 0.0005), we performed a subgroup analysis to investigate the impact of covariates according to the results of the linear mixed model reported by Karlars T et al. [44]. As shown in Table 1 and Additional file 1: Figure S5, the diagnostic performance of CAP was influenced by the geographic regions where the studies were conducted, the cutoff values, age and BMI. Papers from Asian countries suggested a better diagnostic performance and a lower heterogeneity, as compared to those from Europe and USA centers. The diagnostic accuracy of CAP for ≥S2 steatosis was superior with lower heterogeneity when the low cutoff values (below the median values) were used as opposed to the studies where high cutoff values (above the median values) were used. The DOR of CAP for ≥S2 steatosis indicated a better diagnostic performance but high heterogeneity for patients of > 45 years. In contrast, CAP exhibited a high diagnostic performance but a low heterogeneity in patients with BMI 25–30 kg/m^2^.

**Table 1:** Analysis for the efficacy of CAP in the diagnosis of ≥S2 steatosis

Category	Subgroups	Case (n)	AUROC	SE	SP	DOR	*I* ^*2*^
Region	Europe + USA	4	0.7556	0.82 (0.77–0.86)	0.72 (0.65–0.78)	16.80 (4.55–61.99)	77.0%
	Asia	4	0.8798	0.90 (0.83–0.92)	0.76 (0.69–0.81)	27.50 (16.36–46.25)	48.9%
Cutoff value	≥ Median	4	0.7839	0.81 0.76–0.85	0.75 0.69–0.80	13.41 4.93–36.46	77.9%
	< Median	4	0.8799	0.92 0.87–0.95	0.72 0.65–0.79	35.10 17.43–70.67	53.1%
BMI	25–30 kg/m^2^	4	0.8619	0.90 (0.86–0.93)	0.74 (0.68–0.79)	29.88 (17.73–50.38)	44.3%
	> 30 kg/m^2^	3	0.7869	0.79 (0.74–0.84)	0.73 (0.66–0.80)	9.43 (6.04–14.71)	80.5%
Age (mean)	≤ 45 year	3	0.8555	0.86 (0.80–0.91)	0.77 (0.69–0.84)	19.74 (10.95–35.58)	33.0%
	> 45 year	5	0.8143	0.84 (0.80–0.88)	0.73 (0.67–0.78)	23.31 (6.86–79.26)	81.9%

*Abbreviations*: *AUROC* area under the receiver operating characteristic curve, *BMI* body mass index, *DOR* diagnostic odds ratio, *NR* no reported, *SE* sensitivity, *SP* specificity

Using the Leave-One-Out approach, we analyzed the influence of sensitivity of eligible article on the detection of ≥S2 steatosis. It was revealed that following sequential removal of the individual article, the pooled DOR of the included articles fluctuated across arange of confidence interval. As shown in Additional file 1: Figure S6, removing two studies (de Ledinghen V or Park CC) were influenced the DOR value (26.35 and 27.52) obviously, while others changed moderately within the estimated values.

### Publication bias

Based on the Deeks’ Funnel plot (Additional file 1: Figure S7), publication bias was not detected in the studies where CAP was used to detect hepatic steatosis of ≥S1 (*P* = 0.14, Additional file 1: Figure S7, A), ≥S2 (*P* = 0.51, Additional file 1: Figure S7, B), and ≥ S3 (*P* = 0.08, Additional file 1: Figure S7, C).

## Discussion

The extent of hepatic steatosis is closely related to liver-related morbidity and mortality, development of systemic diseases (e.g, cardiovascular disorders) and cancers [45]. Without appropriate intervention, long-term simple fatty liver in NAFLD patients may develop into NASH, liver fibrosis, liver cirrhosis and even hepatocellular carcinoma (HCC) [46, 47].

Currently, accurate assessment of NAFLD is mostly dependent on liver biopsy. However, liver biopsy is an invasive procedure, and it is impractical to conduct this procedure for disease surveillance and progression monitoring. As such, highly accurate and non-invasive surrogate tests for the diagnosis and disease monitoring in NAFLD patients are needed. In this aspect, increasing number of non-invasive approaches for evaluating the extent of fatty liver disease has been explored. Controlled attenuation parameter (CAP) is a convenient examination that correlates the VCTE data with the underlying histological steatosis grades. Its accuracy in evaluating the degree of hepatic steatosis in NAFLD patients has been studied in different patient populations. However, CAP only reflects the proportion of hepatocytes affected by steatosis but it may not provide clues of etiological factors in patients with fatty liver [46].

In this meta-analysis, the diagnostic performance of CAP in different degrees of hepatic steatosis was evaluated in suspected NAFLD patients. Our data revealed that the DOR and AUROC of CAP exhibited a better diagnostic value for hepatic steatosis of Sl and S2 than for the ≥S3 steatosis. Using the sensitivity and specificity of the pooled data, it was revealed that CAP had a low accuracy of identifying severe hepatic steatosis because of high rates of missed or wrong diagnosis of patients with ≥S3 steatosis.

Fagan plot analysis also showed a poor performance of CAP in detecting severe steatosis in suspected NAFLD patients. The poor diagnostic value of CAP for severe steatosis is at least partially due to the thick subcutaneous tissue in the testing subjects, as reported by Shen et al. that the AUROC of CAP was superior in detecting≥S3 steatosis in patients with a skin-to-liver capsular distance (SLCD) of < 25 mm than in the subjects with a SLCD of > 25 mm [48].

Our study has revealed a significant heterogeneity (73.3%) for ≥S2 steatosis. To identify the possible factors that are responsible for heterogeneity and to assess the reliability and stability of this meta-analysis, we used subgroup and sensitivity analysis. Of note, type 2 diabetes and liver fibrosis were not included in the analysis because the covariate data necessary for recalculation could not be extracted from the original studies. Among the factors we analyzed, the geographic regions where the studies were performed, cutoff values used in the included studies, as well as age and BMI were likely responsible for the observed heterogeneity. In fact, the poor accuracy of CAP in the diagnosis and grading of steatosis was previously reported [49, 50]. In this aspect, our current study is consistent with the previously published data that CAP has a limited value in the assessment of hepatic steatosis and this can be attributed to multiple factors as discussed above. In addition, the sensitivity analysis indicated the two studies of de Ledinghen V and Park CC could exist a high risk bias.

In this analysis, we selected highly representative studies and revealed that CAP may not be a reliable test for the detection of moderate to severe steatosis in suspected NAFLD patients. Thus, clinicians should be cautious when using CAP to assess the severity of hepatic steatosis. We also observed that geographic regions where CAP was performed and the cutoff values used by individual centres may contribute to the CAP data inconsistencies, hence further multi-centre and large cohort studies or large population-based studies are needed to validate the clinical application of CAP in the assessment of patients with hepatic steatosis.

It should be noted that our study has several limitations. *Firstly*, the nine studies we analyzed were published in English journals. Thus, potentially high quality articles published in non-English journals might have been missed and some of these studies have limited sample size. In addition, studies showing negative outcomes and poor diagnostic performance might not have been published. Meanwhile, the limited sample size of the included studies may also compromise the data interpretation. Thus, generalizability of our conclusions needs to be further confirmed.

*Secondly*, the data from conventional meta-analysis may not have the same strength as those obtained from multicenter trials because the quality of the meta-analysis may be affected by several factors such as different methodologies used in the studies, study designs, and procedures for analysis. Hence, variations exist between different centers and cohorts. Ideally, most “optimal” cutoff values should be used for more accurate diagnosis, but the so-called “optimal cutoff values” are unable to define. Moreover, diagnostic threshold value may be influenced by natural observation and disease prevalence, resulting in data heterogeneity. Hence, it is difficult to evaluate the diagnostic threshold of CAP with limited number of studies.

*Thirdly*, CAP provides an estimate of what percentage of hepatocytes is affected steatosis whereas the histological evaluation by liver biopsy provides information on the pathological changes in liver parenchyma. Using liver biopsy as the “gold standard” in the assessment of hepatic steatosis may be imperfect, as steatosis may be focal and the sampling error is still a major challenge for liver biopsy [51, 52]. Thus, perfect correlation between CAP and liver biopsy data should not be expected. In addition, since this is a very selected population in which apparently other liver diseases were ruled out and pre-test probability of NAFLD was very high, the diagnostic accuracy of CAP for evaluation of steatosis presence is over-inflated comparing with the general population.

In summary, CAP is a supplementary feature in transient elastography (TE), and it was initially made available for the M probe which has a limited applicability in obese patients [53]. CAP is more suitable for patients with normal or moderate body weight because abdominal fat may increase the skin-to-liver capsule distance by > 25 mm, which could result in overestimation of the CAP data [54]. Such a drawback of CAP in obese patients was tackled by applying XL probe to TE [55], but many authors suggested this approach is not reliable in patients with severe obesity due to the lack of the reliable reference criteria. Hence, the reliability of CAP in the diagnosis and staging of steatosis in suspected NAFLD patients should be further analyzed in large cohort of patients.

## Conclusions

Based on the above analysis, we can conclude that although CAP could be considered as a promising non-invasive test for diagnosing and staging of hepatic steatosis because of its ease of operation and less sampling errors, and it may provide useful guidance to clinicians on whether liver biopsy would be necessary, the diagnostic power of CAP is more superior for ≥S1 steatosis to ≥S2 and ≥ S3 steatosis. When used in patients with ≥S3 steatosis, high rates of missed or wrong diagnosis may occur. Moreover, CAP has a limited utility in obese patients, making its widespread application in patients with metabolic syndrome such as NAFLD a practical concern. Therefore, in clinical practice, the role of CAP as a potential non-invasive substitute for liver biopsy in the assessment of steatosis should be further validated. Searching for non-invasive, inexpensive, and more accurate methods for identifying and staging liver steatosis in suspected NAFLD patients should be a constant endeavour.

## Additional file

Additional file 1: **Figure S1.** Diagnostic odds ratio (DOR) of CAP for steatosis in suspected NAFLD patients. **Figure S2.** Fagan nomogram analysis of the post-test probability of CAP in the detection of ≥ S1 hepatic steatosis (Stage 0 vs Stage 1-3) in suspected NAFLD patients. **Figure S3.** Fagan nomogram analysis of the post-test probability of CAP in the detection of ≥ S2 hepatic steatosis (Stage 0-1 vs Stage 2-3) in suspected NAFLD patients. **Figure S4.** Fagan nomogram analysis of the post-test probability of CAP in the detection of ≥ S3 hepatic steatosis (Stage 0-2 vs Stage 3) in suspected NAFLD patients. **Figure S5.** Subgroup analysis of the diagnostic accuracy of CAP in the detection of ≥ S2 steatosis (Stage 0-1 vs Stage 2-3) in suspected NAFLD patients. **Figure S6.** Analysis of sensitivity of CAP in the diagnosis of ≥ S2 steatosis (Stage 0-1 vs Stage 2-3) in suspected NAFLD patients. **Figure S7.** Estimation of the publication bias by Deek’s Funnel plots. **Table S1.** Basic characteristics of the eligible studies. **Table S2.** Basic statistic analysis of the eligible studies. (DOCX 209 kb)

## Abbreviations

AUROC: Area under the receiver operating characteristic curve
CAP: Controlled attenuation parameter
CI: Confidence interval
DOR: Diagnostic odds ratio
FN: False negative
FP: False positive
LR: Likelihood ratio
NAFLD: Nonalcoholic fatty liver disease
NASH: Non-alcoholic steatohepatitis
QUADAS: Quality assessment of diagnostic accuracy studies
SE: Sensitivity
SP: Specificity
TN: True negative
TP: True positive
