# Supplementary material for: Diagnostic accuracy of controlled attenuation parameter (CAP) as a non-invasive test for steatosis in suspected non-alcoholic fatty liver disease: a systematic review and meta-analysis
Source: BMC Gastroenterol. 2019 Apr 8;19:51. doi: 10.1186/s12876-019-0961-9 (PMC6454693; doi:10.1186/s12876-019-0961-9)
Supplement: Supplementary file 1 — Figure S1. Diagnostic odds ratio (DOR) of CAP for steatosis in suspected NAFLD patients. Figure S2. Fagan nomogram analysis of the post-test probability of CAP in the detection of ≥ S1 hepatic steatosis (Stage 0 vs Stage 1-3) in suspected NAFLD patients. Figure S3. Fagan nomogram analysis of the post-test probability of CAP in the detection of ≥ S2 hepatic steatosis (Stage 0-1 vs Stage 2-3) in suspected NAFLD patients. Figure S4. Fagan nomogram analysis of the post-test probability of CAP in the detection of ≥ S3 hepatic steatosis (Stage 0-2 vs Stage 3) in suspected NAFLD patients. Figure S5. Subgroup analysis of the diagnostic accuracy of CAP in the detection of ≥ S2 steatosis (Stage 0-1 vs Stage 2-3) in suspected NAFLD patients. Figure S6. Analysis of sensitivity of CAP in the diagnosis of ≥ S2 steatosis (Stage 0-1 vs Stage 2-3) in suspected NAFLD patients. Figure S7. Estimation of the publication bias by Deek’s Funnel plots. Table S1. Basic characteristics of the eligible studies. Table S2. Basic statistic analysis of the eligible studies. (DOCX 209 kb) [file 12876_2019_961_MOESM1_ESM.docx]

**Diagnostic accuracy of controlled attenuation parameter (CAP) as a non-invasive test for steatosis in suspected non alcoholic fatty liver disease: a systematic review and metanalysis**

Ke Pu, Yuping Wang, Suyang Bai, Hui Wei, Yongning Zhou, Jiangao Fan, Liang Qiao

**Contents of Supplementary Information**

**Supplementary Figures**

**Figure S1.** Diagnostic odds ratio (DOR) of CAP for steatosis in suspected NAFLD patients. A. DOR for ≥S1 (Stage 0 vs Stage 1-3); B. ≥S2 (Stage 0-1 vs Stage 2-3); C. DOR for ≥S3 (Stage 0-2 vs Stage 3).

**Figure S2.** Fagan nomogram analysis of the post-test probability of CAP in the detection of ≥S1 hepatic steatosis (Stage 0 vs Stage 1-3) in suspected NAFLD patients. **A.** Pre-test probability=25% (patients with the low suspicion for NAFLD). The post-test positive and negative probability of S1 steatosis are 77% and 4%, respectively. **B.** Pre-test probability=50% (patients with the moderate suspicion for NAFLD), the post-test positive and negative probability of S1 steatosis are 91% and 11%, respectively. **C.** Pre-test probability=75% (patients with the high suspicion for NAFLD), the post-test positive and negative probability of S1 steatosis are 97% and 28%, respectively. In each panel of the Fagan plots, three perpendicular axes represent the pre-test probability (left), the likelihood ratio (middle) and the post-test probability (right), respectively.

**Figure S3.** Fagan nomogram analysis of the post-test probability of CAP in the detection of ≥2 hepatic steatosis (Stage 0-1 vs Stage 2-3) in suspected NAFLD patients. **A.** Pre-test probability=25% (patients with the low suspicion for NAFLD), the post-test positive and negative probability of S2 steatosis are 53% and 5%, respectively. **B.** Pre-test probability=50% (patients with the moderate suspicion for NAFLD), the post-test positive and negative probability of S2 steatosis are 77% and 13%, respectively. **C.** Pre-test probability=75% (patients with the high suspicion for NAFLD), the post-test positive and negative probability of S2 steatosis are 91% and 31%, respectively. In each panel of the Fagan plots, three perpendicular axes represent the pre-test probability (left), the likelihood ratio (middle) and the post-test probability (right), respectively.

**Figure S4.** Fagan nomogram analysis of the post-test probability of CAP in the detection of ≥S3 hepatic steatosis (Stage 0-2 vs Stage 3) in suspected NAFLD patients. **A.** Pre-test probability=25% (patients with the low suspicion for NAFLD), the post-test positive and negative probability of S3 steatosis are 39% and 11%, respectively. (B) Pre-test probability=50% (patients with the moderate suspicion for NAFLD), the post-test positive and negative probability of S3 steatosis steatosis are 66% and 27%, respectively. (C) Pre-test probability=75% (patients with the high suspicion for NAFLD), the post-test positive and negative probability of S3 steatosis are 85% and 53%, respectively. The perpendicular axes represent the pre-test probability (left), the likelihood ratio (middle) and the post-test probability (right), respectively.

**Figure S5.** Subgroup analysis of the diagnostic accuracy of CAP in the detection of ≥S2 steatosis (Stage 0-1 vs Stage 2-3) in suspected NAFLD patients. **A.** Regions/countries. **B.** Diagnostic cutoff values. **C.** Body mass index (BMI). **D.** Age of patients

**Figure S6.** Analysis of sensitivity of CAP in the diagnosis of ≥S2 steatosis (Stage 0-1 vs Stage 2-3) in suspected NAFLD patients. No studies can be considered as dominating the overall results or major contributors for study heterogeneity.

**Figure S7.** Estimation of the publication bias by Deek’s Funnel plots.**A.** Analysis on the publications concerning steatosis of ≥S1 steatosis (Stage 0 vs Stage 1-3). **B.** Analysis on the publications concerning ≥S2 steatosis (Stage 0-1 vs Stage 2-3). **C.** Analysis on the publications concerning steatosis of ≥S3 steatosis (Stage 0-2 vs Stage 3). No evidence of publication bias observed.

**Supplementary Tables**

**Table S1.** Basic characteristics of the eligible studies.

**Table S2.** Basic statistic analysis of the eligible studies.

**Figure S1.**


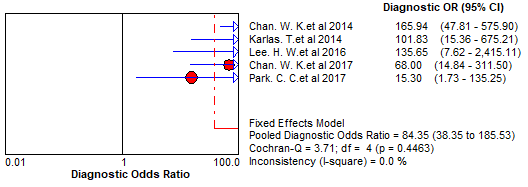


A


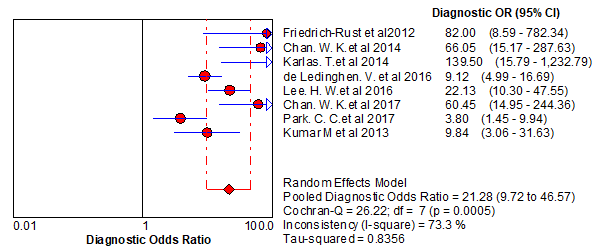


B


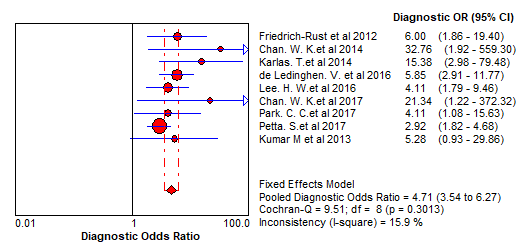


C

**Figure S2.**

C

B

A

**Figure S3.**

C

B

A

**Figure S4.**

A

B

C

**Figure S5.**

A

B

D

C

**Figure S6.**


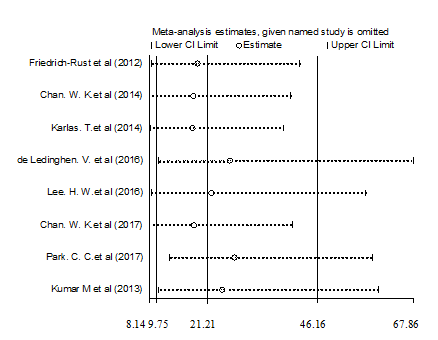


**Figure S7.**

A

B

C

**Table S1.**

| Reference | Region | Sample  (Grade  S1/2/3) | Age  (mean ± SD, y) | Male  (%) | BMI  (Kg/m^2^) | S0 vs S1-3 | | | | S0-1 vs S2-3 | | | | S0-2 vs S3 | | | |
| --- | --- | --- | --- | --- | --- | --- | --- | --- | --- | --- | --- | --- | --- | --- | --- | --- | --- |
|  |  |  |  |  |  | SE  (%) | SP  (%) | Optimal  cutoff  (dB/m) | AUC  value | SE  (%) | SP  (%) | Optimal  cutoff  (dB/m) | AUC  value | SE  (%) | SP  (%) | Optimal  cutoff  (dB/m) | AUC  value |
| Friedrich-Rust et al, 2012 | Germany | 57  (15/18/24) | 45.0±14.0 | 52.6 | 28.0±5.5 | NR | NR | NR | NR | 97.0 | 67.0 | 245.0 | 0.78 | 76 | 68 | 301.0 | 0.72 |
| Chan WK  et al, 2014 | Malaysia | 161  (33/51/14) | 50.3±11.3 | 51.5 | 29.4±3.9 | 91.8 | 93.7 | 263.0 | 0.97 | 96.9 | 67.7 | 263.0 | 0.86 | 100.0 | 53.1 | 281.0 | 0.75 |
| Karlas T  et al, 2014 | Germany | 65  (18/20/12) | 50.4±12.9(S1)  60.0±7.5(S2)  54.7±9.1(S3) | 50.0 | 25.9±4.1(S1)  29.0±4.0(S2)  33.0±4.9(S3) | 93.0 | 87.0 | 233.5 | 0.93 | 97.0 | 81.0 | 268.5 | 0.94 | 82.0 | 76.0 | 301.2 | 0.82 |
| de Ledinghen V et al, 2016 | France | 261  (78/100/83) | 56.1±12.3 | 38.6 | 30.2±5.1 | NR | NR | NR | NR | 79.0 | 71.0 | 310.0 | 0.80 | 87.0 | 47.0 | 311.0 | 0.66 |
| Lee HW  et al, 2016 | Korea | 183  (76/65/33) | 40.6±14.4 | 60.7 | 27.9±4.3 | 88.2 | 100.0 | 247.0 | 0.95 | 84.7 | 80.0 | 280.0 | 0.8 | 72.7 | 60.7 | 300.0 | 0.73 |
| Chan WK  et al, 2017 | Malaysia | 79  (13/28/15) | 50.1±10.4 | 49.4 | 30.2±5.0 | 91.1 | 87.0 | 266.0 | 0.94 | 91.1 | 87.0 | 266.0 | 0.80 | 100.0 | 40.6 | 267.0 | 0.69 |
| Park CC  et al, 2017 | US | 104  (49/29/16) | 50.8±14.6 | 43.3 | 30.4±5.2 | 71.8 | 85.7 | 261.0 | 0.85 | 63.3 | 68.8 | 305.0 | 0.70 | 63.6 | 70.1 | 312.0 | 0.73 |
| Petta S  et al, 2017 | Multicenter | 324  (86/123/115) | 54.2±12.8 | 44.4 | NR | NR | NR | NR | NR | NR | NR | NR | NR | 63.8 | 62.5 | 320.0 | 0.69 |
| Kumar M  et al, 2013 | India | 63  (26/30/7) | NR* | 73.0 | 25.1±2.0 | NR | NR | NR | NR | 78.4 | 73.1 | 258 | 0.79 | 71.4 | 67.9 | 283 | 0.76 |

Abbreviations: BMI: body mass index; SE: Sensitivity; SP: Specificity; AUC: area under the curve; NR: No reported; * midian (range):37 (18-66)

**Table S2.**

| References | S0 vs S1-3 | | | | | | S0-1 vs S2-3 | | | | | | S0-2 vs S3 | | | | | |
| --- | --- | --- | --- | --- | --- | --- | --- | --- | --- | --- | --- | --- | --- | --- | --- | --- | --- | --- |
|  | TP | FP | FN | TN | PPV | NPV | TP | FP | FN | TN | PPV | NPV | TP | FP | FN | TN | PPV | NPV |
| Friedrich-Rust et al, 2012 | NR | NR | NR | NR | NR | NR | 41 | 5 | 1 | 10 | 89.1 | 91.0 | 18 | 11 | 6 | 22 | 62.1 | 78.6 |
| Chan WK et al, 2014 | 90 | 4 | 8 | 59 | 95.7 | 88.1 | 63 | 31 | 2 | 65 | 67.0 | 97.0 | 14 | 69 | 0 | 78 | 16.9 | 100.0 |
| Karlas T et al, 2014 | 47 | 2 | 3 | 13 | 95.9 | 78.8 | 31 | 6 | 1 | 27 | 83.1 | 96.4 | 10 | 13 | 2 | 40 | 43.5 | 95.2 |
| de Ledinghen V et al, 2016 | NR | NR | NR | NR | NR | NR | 145 | 23 | 38 | 55 | 86.3 | 59.1 | 72 | 94 | 11 | 84 | 43.4 | 88.4 |
| Lee HW et al, 2016 | 153 | 0 | 21 | 9 | 100.0 | 30.0 | 83 | 17 | 15 | 68 | 83.0 | 81.9 | 24 | 59 | 9 | 91 | 60.7 | 91.0 |
| Chan WK et al, 2017 | 51 | 3 | 5 | 20 | 94.4 | 80.0 | 39 | 5 | 4 | 31 | 88.6 | 88.6 | 15 | 38 | 0 | 26 | 28.3 | 100.0 |
| Park CC et al, 2017 | 51 | 1 | 20 | 6 | 98.1 | 23.1 | 19 | 15 | 11 | 33 | 55.9 | 75.0 | 7 | 20 | 4 | 47 | 25.9 | 92.2 |
| Petta S et al, 2017 | NR | NR | NR | NR | NR | NR | NR | NR | NR | NR | NR | NR | 73 | 78 | 42 | 131 | 48.3 | 75.7 |
| Kumar M et al, 2013 | NR | NR | NR | NR | NR | NR | 29 | 7 | 8 | 19 | 80.6 | 70.4 | 5 | 18 | 2 | 38 | 21.7 | 95.0 |

Abbreviations: TP: True positive; FP: False positive; FN: False negative; TN: true negative; PPV: Positive prediction value; NPV: Negative prediction value; NR: No reported.
